# m^6^A RNA methylation drives kidney fibrosis by upregulating β-catenin signaling

**DOI:** 10.7150/ijbs.96233

**Published:** 2024-06-03

**Authors:** Yinyi Long, Dongyan Song, Liuyan Xiao, Yadie Xiang, Dier Li, Xiaoli Sun, Xue Hong, Fan Fan Hou, Haiyan Fu, Youhua Liu

**Affiliations:** 1 State Key Laboratory of Organ Failure Research, National Clinical Research Center of Kidney Disease, Division of Nephrology, Nanfang Hospital, Southern Medical University, Guangzhou, China.; 2 Guangdong Provincial Key Laboratory of Renal Failure Research, Guangdong Provincial Institute of Nephrology, Guangzhou, China.

**Keywords:** METTL3, m^6^A modification, β-catenin, IGF2BP3, kidney fibrosis, chronic kidney disease

## Abstract

N^6^-methyladenosine (m^6^A) methylation plays a crucial role in various biological processes and the pathogenesis of human diseases. However, its role and mechanism in kidney fibrosis remain elusive. In this study, we show that the overall level of m^6^A methylated RNA was upregulated and the m^6^A methyltransferase METTL3 was induced in kidney tubular epithelial cells in mouse models and human kidney biopsies of chronic kidney disease (CKD). Proximal tubule-specific knockout of METTL3 in mice protected kidneys against developing fibrotic lesions after injury. Conversely, overexpression of METTL3 aggravated kidney fibrosis *in vivo*. Through bioinformatics analysis and experimental validation, we identified β-catenin mRNA as a major target of METTL3-mediated m^6^A modification, which could be recognized by a specific m^6^A reader, the insulin-like growth factor 2 mRNA binding protein 3 (IGF2BP3). METTL3 stabilized β-catenin mRNA, increased β-catenin protein and induced its downstream profibrotic genes, whereas either knockdown of IGF2BP3 or inhibiting β-catenin signaling abolished its effects. Collectively, these results indicate that METTL3 promotes kidney fibrosis by stimulating the m^6^A modification of β-catenin mRNA, leading to its stabilization and its downstream profibrotic genes expression. Our findings suggest that targeting METTL3/IGF2BP3/β-catenin pathway may be a novel strategy for the treatment of fibrotic CKD.

## Introduction

Chronic kidney diseases (CKD), characterized by decline of kidney function and progressive tissue fibrosis, are highly prevalent worldwide 1. CKD carries a substantial risk of progressing to end-stage renal failure, a devastating condition with high mortality and requiring renal replacement therapy 2. Kidney fibrosis is the common outcome of virtually all CKD, irrespective of the initial causes 3. The pathophysiology of kidney fibrosis is complex and involves various types of cells 4, 5. As the principal component of renal parenchyma, tubular epithelial cells are particularly vulnerable to injury and respond in different ways including partial epithelial‐mesenchymal transition, metabolic reprogramming, cellular senescence and cell cycle arrest 6-12. Regulation of these responses involves the engagement of several key signal pathways, including Wnt/β-catenin signaling.

Wnt/β-catenin is an evolutionarily conserved signal cascade and plays a fundamental role in kidney development, injury repair and fibrosis 13. Mounting evidence indicates that sustained activation of Wnt/β-catenin contributes to kidney fibrosis 14, 15, whereas blockade of this signaling ameliorates fibrotic lesions in CKD 16-18. It is widely presumed that β-catenin, the principal mediator of Wnt signaling, is mainly controlled at the posttranslational level through phosphorylation and ubiquitination-mediated degradation 19. However, we recently uncovered that insulin-like growth factor 2 mRNA-binding protein 3 (IGF2BP3) is involved in the regulation of β-catenin mRNA stability, suggesting that β-catenin is also controlled at the posttranscriptional level 20. Of interest, IGF2BP3 is known to be involved in the RNA N^6^-methyladenosine (m^6^A) modification as one of the methylation recognition proteins 21. These findings prompted us to speculate that m^6^A RNA methylation may play a role in regulating β-catenin expression in the pathogenesis of fibrotic CKD.

m^6^A methylation is a common modification in mammalian mRNA and plays critical roles in various biological processes and the pathogenesis of human diseases 22, 23. m^6^A methylation regulates many aspects of RNA trajectory and fate, including RNA stability, degradation, transport, splicing and translation 24. The process of m^6^A methylation is reversible and dynamic, controlled by three types of protein complexes including RNA methyltransferases (writers), demethylases (erasers) and methylation recognition proteins (readers) 25. m^6^A is placed on mRNA by a methyltransferase complex composed primarily of methyltransferase-like 3 (METTL3), METTL14 and Wilms tumor 1-associating protein (WTAP). METTL3 is the catalytically active methyltransferase and METTL14 plays a role in substrate recognition, while WTAP ensures the METTL3-METTL14 heterodimer is localized to nuclear speckles to facilitate catalysis 26, 27. As the major catalytic subunit in m^6^A methyltransferase complex, METTL3 is known to play a pivotal role in generating m^6^A modification. However, its role and mechanism of action in the evolution of kidney fibrosis remains poorly understood.

In this study, we demonstrate that overall m^6^A methylation is upregulated in mouse models of kidney fibrosis and METTL3 is upregulated in human CKD and correlates with the severity of CKD. Conditional knockout of METTL3 in proximal tubules alleviates kidney fibrotic lesions, while overexpression of METTL3 exacerbates renal injury. Furthermore, we have identified β-catenin mRNA as a target of METTL3-mediated m^6^A modification. These studies suggest that METTL3-mediated m^6^A modification could be a therapeutic target for treating fibrotic CKD.

## Methods

### Animal models

All animal studies were approved by the Animal Ethics Committee at the Nanfang Hospital (NFYY-2020-0953). Male C57BL/6 mice were obtained from the Experimental Animal Center of Southern Medical University. Unilateral ischemia-reperfusion injury (UIRI) and unilateral ureteral obstruction (UUO) models were established according to routine protocols 8, 28, 29.

The METTL3^flox/flox^ mice were generated by GemPharmatech (Nanjing, China). The proximal tubule-specific conditional knockout (cKO) mice were generated by mating METTL3^flox/flox^ mice with phosphoenolpyruvate carboxykinase Cre (PEPCK^Cre^) mice (kindly provided by Dr. Volker Haase, Vanderbilt University) 30. Littermates of cKO (genotype: METTL3^fl/fl^, Cre^+/-^) and wild-type (WT) (genotype: METTL3^fl/fl^) mice were used.

Overexpression of METTL3 *in vivo* was carried out by the hydrodynamics-based gene transfer approach 31. Briefly, mice were injected with empty vector (pcDNA3) or Flag-tagged METTL3 expression vector (pFlag-METTL3) at 2 days after UUO or 4 days after UIRI, respectively. The expression of transgene was validated by Western blotting or immunostaining for Flag-tagged METTL3 fusion protein.

### Human kidney biopsy samples

Human kidney samples were obtained from diagnostic renal biopsies performed at the Nanfang Hospital, with written informed consent from the patients. Human normal kidney controls were obtained from non-tumorous renal tissues of patients who had renal cell carcinoma and underwent nephrectomy. Paraffin-embedded human kidney biopsies sections (3 µm) were prepared and used for immunohistochemical staining. Quantification was assessed by a computer-aided point-counting technique. Studies involving human samples were approved by the Medical Ethics Committee at the Nanfang Hospital (EFEC-2021-051).

### Dot blot assay

RNA isolation was performed using TRIzol reagent (Invitrogen). Total RNA (400 ng) was spotted onto a nylon membrane (Sigma-Aldrich). The membranes were then cross-linked under ultraviolet (UV) light and blocked with 5% nonfat milk at room temperature for 1 h, followed by incubating overnight with the m^6^A antibody (#202003; Synaptic Systems, Goettingen, Germany) and horseradish peroxidase (HRP)-conjugated secondary antibody. After washing, signals were detected using SuperEnhanced chemiluminescence detection reagents and Kodak X-ray film. The amount of total RNA loaded was assessed using 0.02% methylene blue in 0.3 M sodium acetate (pH 5.2).

### MeRIP-qPCR

The m^6^A modifications on specific genes were analyzed using m^6^A RNA enrichment kit (P-9018; Epigentek, Farmingdale, NY) according to the manufacturer's instructions. Briefly, the m^6^A-containing target fragments were pulled down using a bead-bound m^6^A capture antibody, and the RNA sequence encompassing the m^6^A region was cleaved using a lyase cocktail. The enriched RNA was then released, purified, and eluted. The m^6^A RNA levels were assessed by qPCR and quantified after normalizing to the input.

### Cell culture and treatment

Human kidney proximal tubular cells (HK-2) were purchased from the American Type Culture Collection (ATCC) (Manassas, VA). HK-2 cells were transfected with control or METTL3-specific siRNA, followed by incubating with TGF-β1 (4 ng/ml) for another 2 days after serum starvation overnight. For the co‐transfection studies, the METTL3-overexpression plasmid and IGF2BP3-specific siRNA were co‐transfected for 24 h in HK-2 cells. In some experiments, HK-2 cells were treated with ICG-001 (HY-14428; MedChemExpress, Monmouth Junction, NJ) at 10 µM for 48 h, followed by transfection with METTL3-overexpression plasmids. Cells were then collected and subjected to various analyses.

Primary tubular epithelial cells were isolated from the kidneys of cKO and WT mice using an established method 8. In brief, the kidneys were digested with pre-warmed collagenase type IV solution (Invitrogen, USA) for 40 min at 37 °C. The tubules were then centrifuged using 32% Percoll gradients for 10 min. The cells were seeded on the plates in DMEM/F12 medium supplemented with 1% penicillin-streptomycin and 10% fetal bovine serum (FBS).

### Western blot analyses

The procedures of Western blot analysis were described previously 32. Briefly, homogenates from mouse kidneys or cultured cells were prepared. The protein concentration was determined using the BCA assay (Cat. ab287853; Abcam). Protein samples were reduced, denatured, and subjected to Western blot analysis with specific antibodies. SuperEnhanced chemiluminescence detection reagents were used for protein band detection. Relative protein levels were analyzed using ImageJ software and the ratio of specific proteins to GAPDH or α-tubulin was calculated, respectively. Fold induction over the control group (set as 1.0) was reported. Antibodies used are presented in Supplementary Table S1.

### Quantitative real-time RT-PCR

RNA expression was evaluated using qRT-PCR as described 20. The mRNA levels of different genes were assessed after normalized to GAPDH. The sequences of PCR primers are provided in Supplementary Table S2.

### Bioinformatics analyses

Advanced Heatmap Plot of RNA-seq (GSE118339) was performed using the OmicStudio tools at https://www.omicstudio.cn. R studio (version 4.2.2) was used to do gene function enrichment. The intersection gene set was collected from published MeRIP-seq (GSE182607) and RIP-seq (GSE90639). The ClusterProfiler (version 4.6.2) was used to do KEGG and GO enrichment analysis 33-36. *P* value cutoff was set to 0.05.

### Histology, immunohistochemical and immunofluorescence staining

Paraffin-embedded kidney sections from human and mouse tissues were prepared and subjected to Sirius red staining. Immunohistochemical staining were carried out as previously described 37. Antibodies used are listed in Supplementary Table S1.

### *In situ* hybridization

The procedures of *in situ* hybridization (ISH) were described previously 38. The probes for *CTNNB1* (5'-ACTCAAGCTGATTTGATGGAGTTGGACATG-3', 5'-GGGTTCAGATGATATAAATGTGGTCACCTG-3', and 5'-TGCCTCCAGGTGACAGCAATCAGCTGGCCT-3') were purchased from Boster Biological Technology (Wuhan, China).

### *CTNNB1* mRNA stability

To evaluate the mRNA stability of *CTNNB1*, HK-2 cells were transfected with pcDNA3 or METTL3-overexpression plasmids. Cells were then treated with actinomycin D (HY-17559; MedChem Express, Monmouth Junction, NJ) at 5 µg/ml. After incubation for various periods of time as indicated, cells were collected and RNA isolated for qRT-PCR.

### Statistics

The quantitative data were presented as mean ± SEM. Statistical analyses were conducted using IBM SPSS statistical software. For comparisons between two groups, a *t*-test was employed. One-way ANOVA was used for comparisons involving three or more groups. Spearman's correlation analysis (nonparametric) was used to analyze the relationship between METTL3 and other parameters of kidney. *P* < 0.05 was considered significant.

## Results

### The m^6^A RNA methylation is upregulated in mouse models of kidney fibrosis

**Figure 1:**
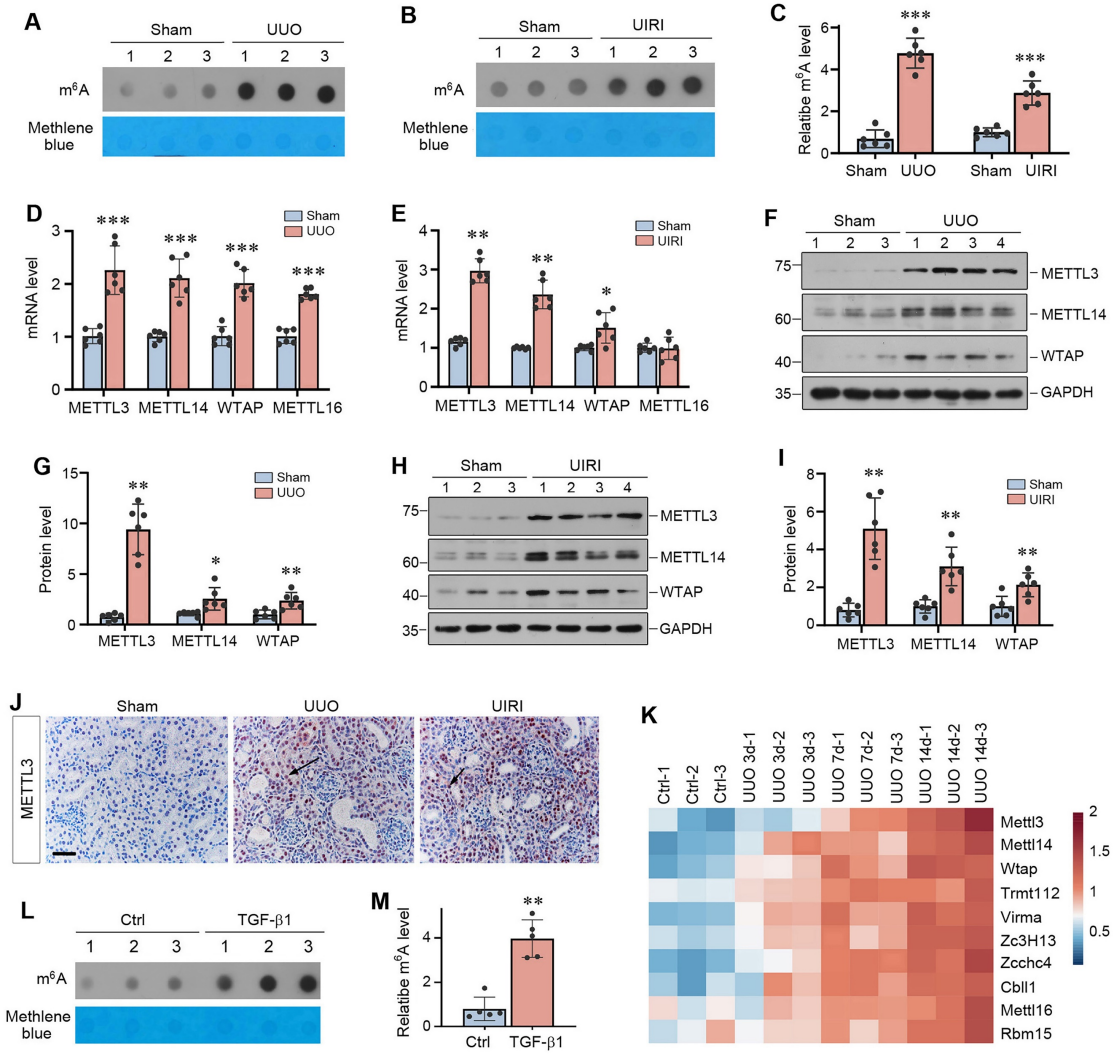
** m^6^A modification is globally upregulated in mouse models of chronic kidney disease (CKD).** (A-C) Dot blot assay (A, B) and quantitative determination (C) of global m^6^A abundances in normal and CKD mice induced by either unilateral ureteral obstruction (UUO) at 7 days (A, C) or unilateral ischemic-reperfusion injury (UIRI) at 11 days (B, C). Methylene blue staining was used as loading control.^ ***^P < 0.001 versus sham (n=6). (D-E) qRT-PCR showed mRNA levels of METTL3, METTL14, and WTAP in the fibrotic kidneys induced by UUO at 7 days (D) or UIRI at 11 days (E), respectively. ^*^P < 0.05, ^**^P < 0.01, ^***^P < 0.001 versus sham (n=6). (F-G) Representative Western blot (F) and quantitative determination (G) of METTL3, METTL14 and WTAP proteins in the kidney after UUO. ^*^P < 0.05, ^**^P < 0.01 versus sham (n=6). (H, I) Representative Western blot (H) and quantitative determination (I) of METTL3, METTL14 and WTAP proteins in the kidney after UIRI. ^**^P < 0.01 versus sham (n=6). (J) Representative micrographs demonstrate the expression and localization of METTL3 protein in normal and diseased kidneys. Arrows indicated positive staining. Scale bar, 50 µm. (K) Re-analysis of the RNA-seq data (GSE118339) demonstrated the dynamics of 10 m^6^A writers in control and UUO kidneys at different time points (day 3, 7, 14) after surgery. Red indicates a higher abundance and blue indicates a lower abundance. (L-M) Dot blot assay (L) and quantitative determination (M) of m^6^A abundance in human proximal tubular cells (HK-2) after treatment with TGF-β1. Methylene blue staining was used as loading control.^ **^P < 0.01 versus Ctrl (n=6).

To investigate the role of m^6^A RNA methylation in kidney fibrosis, we first assessed the overall level of m^6^A methylated RNA in whole kidney lysates of normal and CKD mice by dot blot assay. As shown in Figure 1A-C, a significant increase in m^6^A RNA modifications was observed in the kidneys after UUO and UIRI, suggesting that kidney fibrosis was associated with a global upregulation of m^6^A RNA modification.

We next examined the expression of methyltransferases, the so-called writers of m^6^A RNA methylation. As shown in Figure 1D and E, the mRNA levels of METTL3, METTL14 and WTAP were up-regulated in both UUO at 7 days and UIRI at 11 days, whereas METTL16 was only induced in UUO model. We further examined the protein levels of these methyltransferases by Western blot analysis of whole kidney lysates. As shown in Figure 1F-I, METTL3, METTL14 and WTAP proteins were also induced in the kidneys of UUO and UIRI mice, compared to sham controls. Immunohistochemical staining revealed that METTL3 was predominantly localized in the nuclei of renal tubular cells in UUO and UIRI mice (Figure 1J). In addition, by re-analyzing the data independently obtained from RNA-seq (GSE118339), we found that renal METTL3, METTL14, WTAP and METTL16 were up-regulated at different time points after UUO (Figure 1K), further strengthening our findings. Interestingly, many m^6^A methylation readers and erasers were also upregulated in the fibrotic kidney after UUO (Supplementary Figure S1A). *In vitro*, when human proximal tubular cells (HK-2) were incubated with TGF-β1, m^6^A methylation in these cells was also upregulated (Figure 1L and M). TGF-β1 also induced METTL3 expression in HK-2 cells, which was dependent on Smad3 signaling, as Smad3 inhibitor SIS3 blocked METTL3 induction by TGF-β1 (Supplementary Figure S1B and C). Notably, as METTL3 was the most upregulated methyltransferase in fibrotic kidneys (Figure 1G), we decided to focus on METTL3 in the subsequent studies.

### METTL3 is upregulated in human CKD and associated with kidney dysfunction

**Figure 2:**
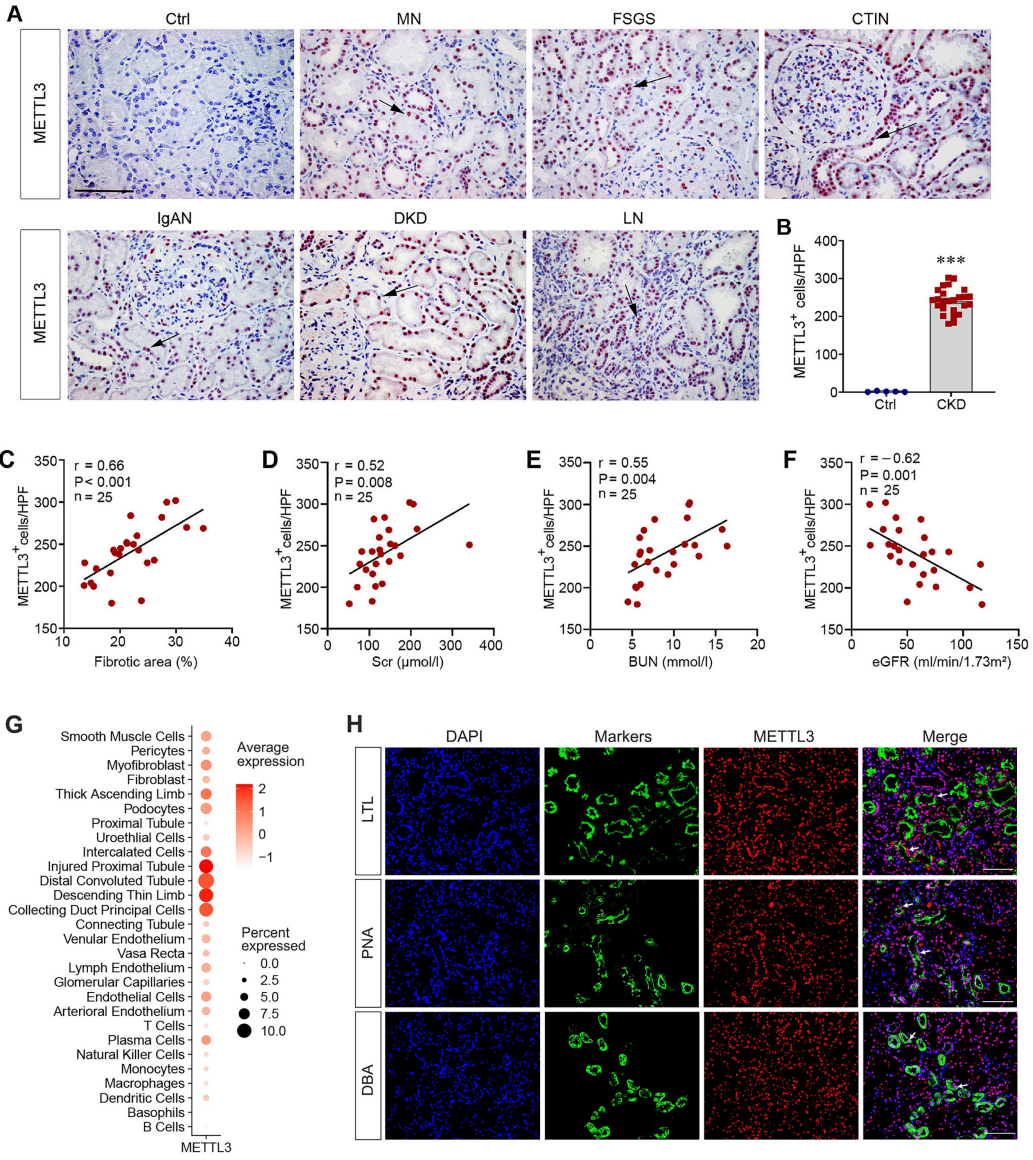
** METTL3 is upregulated in human CKD.** (A) Representative micrographs demonstrate the expression and localization of METTL3 protein in kidney biopsy specimens from CKD patients. Arrows indicate positive staining. Scale bar, 50 µm. Ctrl, non-tumorous adjacent kidney tissue specimens from patients with renal cell carcinoma; DN, diabetic nephropathy; LN, lupus nephritis; IgAN, immunoglobulin A nephropathy; FSGS, focal and segmental glomerulosclerosis; MN, membranous nephropathy; CTIN, chronic tubulointerstitial nephritis. (B) Semi-quantitative determination showed the METTL3 positive cells per high-power field in different groups. ^***^P < 0.001 versus controls (n=5-25). (C-F) Linear regression showed close correlation between METTL3 positive cells per high power field (HPF) and fibrotic area (C), serum creatinine level (Scr) (D), blood urea nitrogen (BUN) (E) and estimated glomerular filtration rate (eGFR) (F), respectively. (G) Reanalysis of single-cell RNA sequencing data (https://zenodo.org/record/4059315) illustrated METTL3 expression in different cell types in CKD kidney. The vertical axis (Y-axis) of this plot represents distinct human kidney cell types featured in the dataset, the intensity of dot color shows the average expression of METTL3, while the dot size shows the relative proportion of METTL3 expression within this dataset. (H) Representative double immunofluorescence staining for METTL3 and tubule segment-specific markers in kidney tissue sections of CKD patients, respectively. Lotus tetragonolobus lectin (LTL) was used to label the proximal tubule; Peanut agglutinin (PNA) was used to label the distal tubule; Dolichos biflorus agglutinin (DBA) was used to label the collecting duct. Arrows indicate positive staining. Scale bar = 100 µm.

To evaluate the clinical relevance of the aforementioned findings, we examined the expression of METTL3 in kidney biopsies from CKD patients by immunohistochemical staining. METTL3 protein was undetectable in the non-tumorous adjacent normal kidney tissue from patients with renal cell carcinoma (Figure 2A). In contrast, substantial expression of METTL3 was evident in kidney biopsies from patients with different CKDs, including membranous nephropathy (MN), focal and segmental glomerulosclerosis (FSGS), chronic tubulointerstitial nephritis (CTIN), IgA nephropathy (IgAN), diabetic kidney disease (DKD) and lupus nephritis (LN) (Figure 2A). METTL3 protein was mainly localized in the nuclei of renal tubular epithelium in CKD patients (Figure 2A, arrows). The relative abundances of METTL3 in control and CKD patients are presented in Figure 2B. The clinical and demographic data of the 25 cases of CKD patients are shown in Supplementary Table S3.

We next explored the connection between METTL3 expression, fibrotic lesions, and kidney function in these patients. There was a close correlation between METTL3 levels and the extent of fibrotic lesions in CKD patients (Figure 2C). Consistently, METTL3 was also associated with serum creatinine (Scr) and blood urea nitrogen (BUN) levels (Figure 2D and E). Moreover, an inverse relationship between METTL3 and estimated glomerular filtration rate (eGFR) was evident in CKD patients (Figure 2F).

To further assess METTL3 expression and localization in human CKD, we re-analyzed a published single-cell transcript sequencing data obtained from the Zenodo data archive (https://zenodo.org/record/4059315). As shown in Figure 2G, METTL3 was highly expressed in the injured proximal tubule, distal tubule and collecting duct in human CKD. We also performed double immunofluorescence staining for METTL3 and tubular segment-specific markers. As shown in Figure 2H, METTL3 was co-expressed with Lotus tetragonolobus lectin (LTL) (proximal tubule marker), Peanut agglutinin (PNA) (distal tubule marker) and Dolichos biflorus agglutinin (DBA) (collecting duct marker) in the kidney of CKD patients, suggesting that METTL3 is expressed in the major segments of kidney tubules in CKD.

### Proximal tubule-specific ablation of METTL3 protects kidney against fibrosis in mice

**Figure 3:**
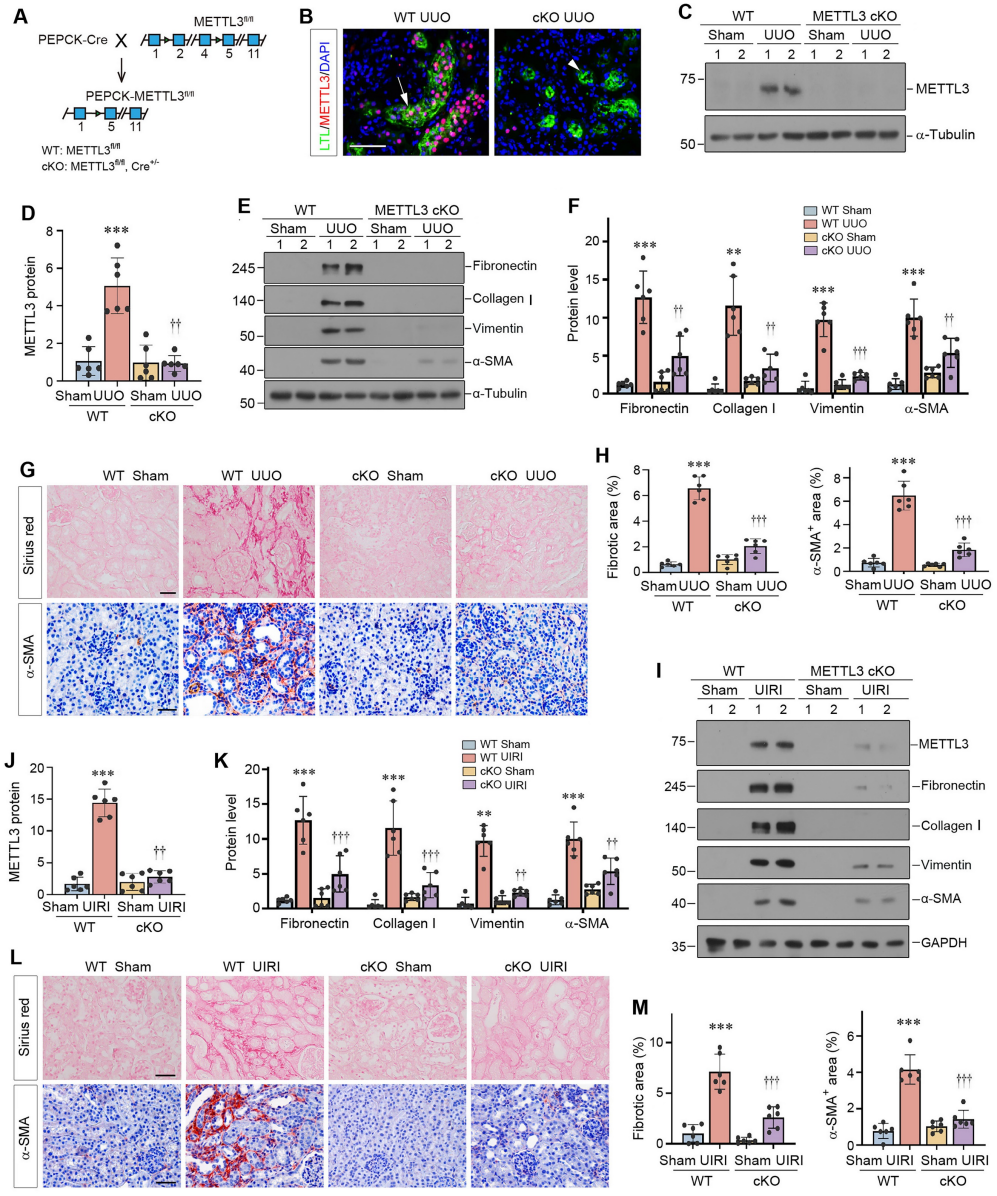
** Proximal tubule-specific conditional knockout of METTL3 protects against kidney fibrosis.** (A) Schematic illustration shows the strategy to generate proximal tubule-specific, conditional METTL3 knockout mice (METTL3 cKO). (B) Double staining of METTL3 and proximal tubular marker LTL in the WT and METTL3-cKO mice at 7 days after UUO. Scale bars, 100 µm. (C-D) Representative Western blot (C) and quantitative data (D) show the expression of METTL3 protein in different groups as indicated. ^***^P < 0.001 versus Sham group; ^††^*P* <0.01 versus WT UUO group (n=6). (E-F) Representative Western blots (E) and quantitative data (F) show the expression of fibronectin, collagen I, vimentin and α-SMA proteins in different groups as indicated. ^**^P < 0.01, ^***^P < 0.001 versus Sham group; ^††^*P* < 0.01, ^†††^*P* < 0.001 versus WT UUO group (n=6). (G-H) Representative micrographs (G) and semi-quantification (H) show collagens deposition by Sirius red staining and α-SMA expression at 7 days after UUO in different groups. ^***^P < 0.001 versus Sham group; ^†††^*P* < 0.001 versus WT UUO group (n=6). Scale bar, 50 µm. (I-K) Representative Western blots (I) and quantitative data (J, K) show the expression of METTL3, fibronectin, collagen I, vimentin and α-SMA proteins in different groups as indicated. ^**^P < 0.01, ^***^P < 0.001 versus Sham group; ^††^*P* < 0.01, ^†††^*P* < 0.001 versus WT UIRI group (n=6). (L-M) Representative micrographs (L) and their semi-quantification (M) show collagens deposition and α-SMA at 11 days after UIRI in different groups. ^***^P < 0.001 versus Sham group; ^†††^*P* < 0.001 versus WT UIRI group (n=6). Scale bar, 50 µm.

To assess the function of METTL3 *in vivo*, we generated a conditional knockout mouse model (METTL3-cKO) with proximal tubule-specific ablation of METTL3 by Cre-loxP system (Figure 3A). Deletion of METTL3 was verified by immunofluorescence staining and Western blotting (Figure 3B-D). These cKO mice and WT (genotype: METTL3^fl/fl^) controls were subjected to UUO for 7 days. As shown in Figure 3E and F, renal expression of fibronectin, collagen Ⅰ, vimentin and α-smooth muscle actin (α-SMA) was inhibited in the obstructed kidney of METTL3 cKO mice, compared to WT controls. Similar results were obtained when kidney sections were subjected to immunostaining for α-SMA (Figure 3G and H) and fibronectin (Supplementary Figure S2A and B). Sirius red staining also showed that deletion of METTL3 ameliorated renal collagen accumulation and fibrotic lesions after UUO (Figure 3G and H).

We expanded these studies by using UIRI model. As shown in Figure 3I-K, renal expression of METTL3, fibronectin, collagen Ⅰ, vimentin and α-SMA were induced in WT mice after UIRI, which was largely abolished by deletion of METTL3 in the cKO mice. Immunostaining for α-SMA and Sirius red staining for collagens further validated this result (Figure 3L and M).

### Overexpression of METTL3 promotes kidney fibrosis after injury *in vivo*

**Figure 4:**
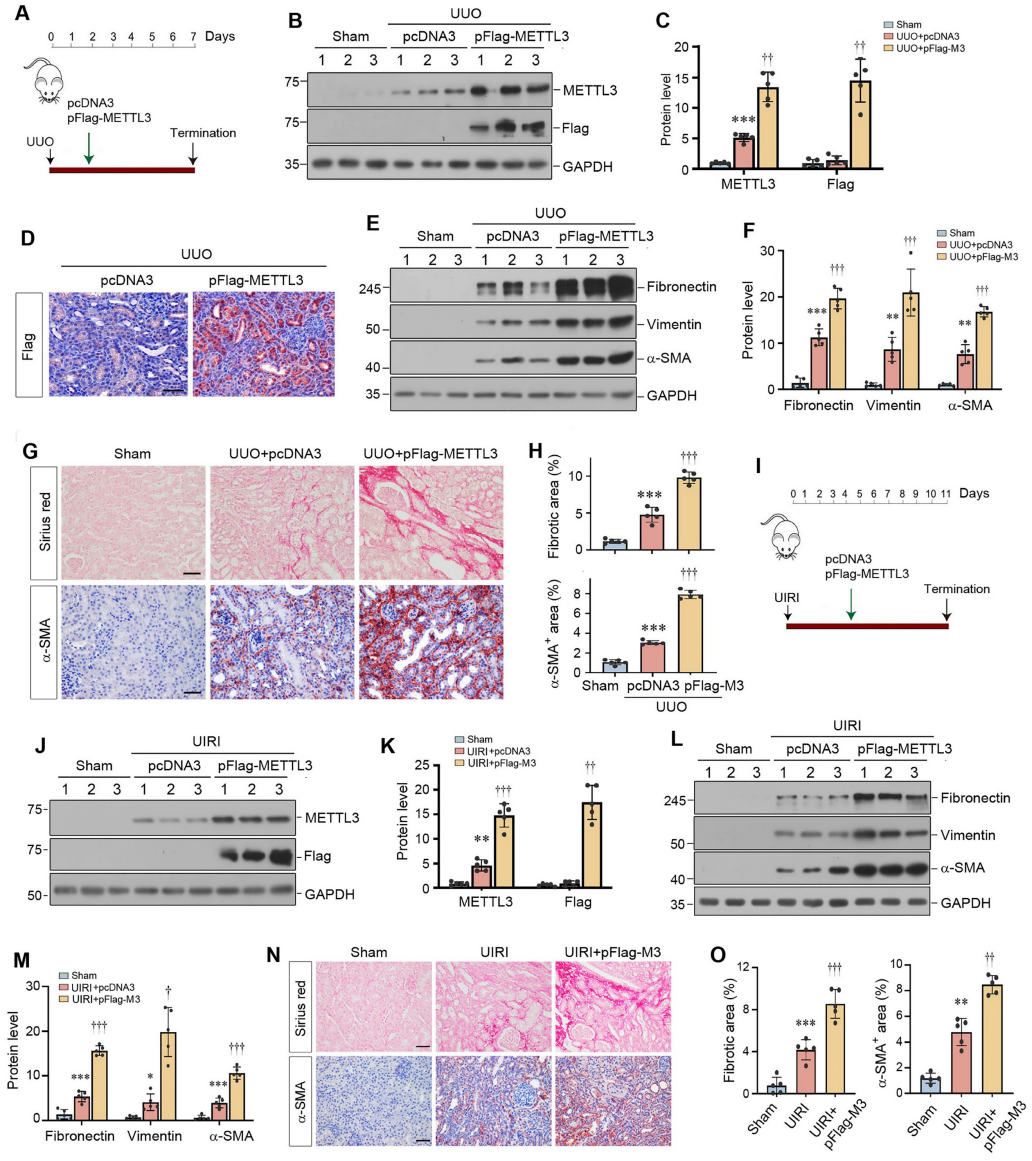
** Overexpression of METTL3 aggravates kidney fibrosis after kidney injury.** (A) Experimental design. The green arrow indicates the timing of injecting pcDNA3 or Flag-tagged METTL3 expression plasmid (pFlag-METTL3). The black arrow indicates the timing of UUO surgery. (B, C) Representative Western blot (B) and quantitative data (C) show the expression of Flag and METTL3 in different groups as indicated. ^***^P < 0.001 versus Sham group; ^††^*P* < 0.01 versus UUO injected with pcDNA3 group (n=5). (D) Micrographs show Flag expression in UUO kidney. Scale bar, 50 µm. (E, F) Representative Western blot (E) and quantitative data (F) show the expression of fibronectin, vimentin and α-SMA proteins in different groups as indicated. ^**^P < 0.01 versus Sham group, ^††^*P* < 0.01, ^†††^*P* < 0.001 versus the UUO injected with pcDNA3 group (n=5). (G, H) Representative micrographs (G) and semi-quantification (H) show the expression of collagens and α-SMA at 7 days after UUO in different groups. Scale bar, 50 µm. ^***^P < 0.001 versus sham group, ^†††^*P* < 0.001 versus UUO injected with pcDNA3 group (n=5). (I) Experimental design. The green arrow indicates the timing of injecting pcDNA3 or pFlag-METTL3. The black arrow indicates the timing of UIRI surgery. (J, K) Representative Western blot (J) and quantitative data (K) demonstrate the expression of Flag and METTL3 in various indicated groups. ^**^P < 0.01 versus Sham group; ^††^*P* < 0.01, ^†††^*P* < 0.001 versus UIRI injected with pcDNA3 group (n=5). (L, M) Representative Western blot (L) and quantitative data (M) illustrate the expression of fibronectin, vimentin and α-SMA proteins in different groups as indicated. ^*^P < 0.05, ^***^P < 0.001 versus Sham group; ^†^*P* < 0.05, ^†††^*P* < 0.001 versus UIRI injected with pcDNA3 group (n=5). (N, O) Representative micrographs (N) and semi-quantification (O) show the expression of collagens and α-SMA at 11 days after UIRI in different groups. ^**^P < 0.01, ^***^P < 0.001 versus Sham group; ^††^*P* < 0.01, ^†††^*P* < 0.001 versus UIRI injected with pcDNA3 group (n=5). Scale bar, 50 µm.

To further confirm a role for METTL3-mediated m^6^A methylation in kidney fibrosis, we decided to overexpress exogenous METTL3 in the kidney *in vivo*. Figure 4A shows the experimental design. Mice were injected with either pcDNA3 or Flag-tagged METTL3 expression plasmid (pFlag-METTL3) at 2 days after UUO by a hydrodynamics-based gene delivery approach 31. Western blot analysis of whole kidney lysates revealed that injection of pFlag-METTL3 resulted in overexpression of Flag-tagged METTL3 in the obstructed kidney (Figure 4B and C). Immunostaining for Flag provided further confirmation of this result (Figure 4D). The Flag-METTL3 fusion protein was predominantly localized in renal tubular epithelium of the obstructed kidney after UUO (Figure 4D).

We found that overexpression of exogenous METTL3 caused an increase in renal expression of fibronectin, vimentin and α-SMA after UUO (Figure 4E and F). Immunostaining further confirmed that METTL3 increased renal expression of α-SMA (Figure 4G and H) and fibronectin (Supplementary Figure S2C and D) in the fibrotic kidney. Sirius red staining also revealed an aggravated deposition of collagens in the obstructed kidney injected with pFlag-METTL3, compared to pcDNA3 (Figure 4G and H). Similarly, injection of pFlag-METTL3 at 4 days after UIRI led to overexpression of Flag-METTL3 fusion protein in the kidney (Figure 4I-K). As shown in Figure 4L and M, METTL3 overexpression aggravated renal expression of fibronectin, vimentin and α-SMA in the kidney after UIRI. Similar results were obtained when kidney sections were stained for α-SMA and collagens (Figure 4N and O).

Consistent with *in vivo* data, overexpression of METTL3 in cultured HK-2 cells also induced fibronectin and vimentin expression (Supplementary Figure S3A and B). METTL3 also induced the expression of p21, a marker of cellular senescence (Supplementary Figure S3A and B).

### Identification of β-catenin mRNA as a target of METTL3-mediated m^6^A modification

We next explored the mechanism of m^6^A modification in regulating kidney fibrosis by identifying its target. Along this line, we previously demonstrated that IGF2BP3, one of the m^6^A readers, is increased in CKD and plays a critical role in kidney fibrosis by activating β-catenin through binding to and stabilizing its mRNA 20. This observation prompted us to speculate that METTL3 might promote kidney fibrosis by methylating β-catenin mRNA, which is then recognized by IGF2BP3.

**Figure 5:**
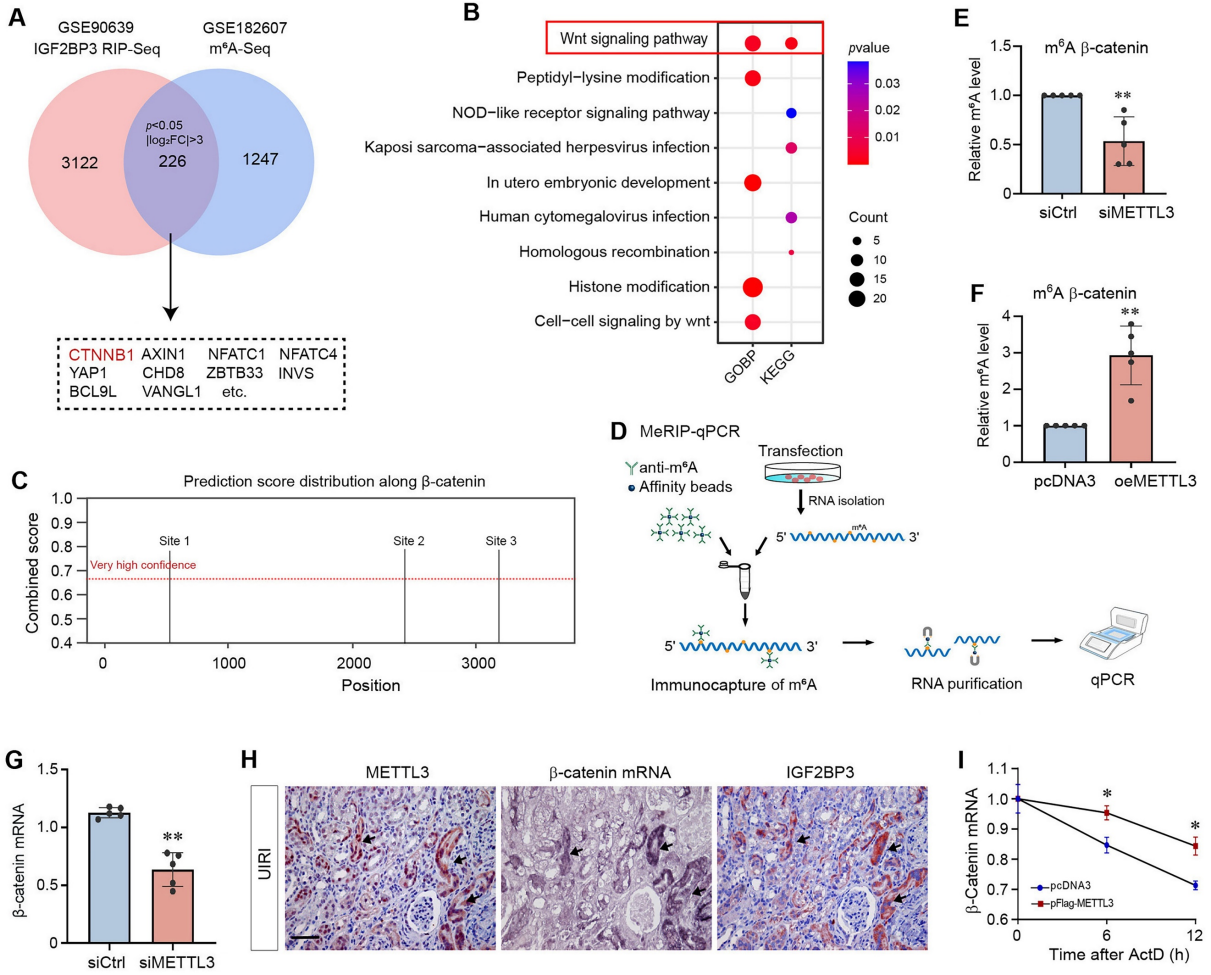
** Identification of β-catenin mRNA as a target of METTL3-mediated m^6^A modification in fibrotic kidney.** (A) Venn diagram shows overlap of the target genes of MeRIP-seq (GSE182607) and IGF2BP3 RIP-seq (GSE90639). (B) The KEGG and GO biological process enrichment of overlap genes. (C) SRAMP analysis of potential m^6^A methylation modification sites on β-catenin mRNA. (D) Diagram shows the MeRIP-qPCR protocol detecting the m^6^A methylated-β-catenin mRNA abundances. (E) Knockdown of METTL3 in HK2 cells decreased m6A methylated β-catenin mRNA detected by MeRIP-qPCR. ^**^P < 0.01 versus the siCtrl group (n=5). (F) Overexpression of METTL3 increased the m6A methylated β-catenin mRNA detected by MeRIP-qPCR. ^**^P < 0.01 versus the pcDNA3 group (n=5). (G) qRT-PCR analyses show that knockdown of METTL3 decreased the steady-state level of β-catenin mRNA in HK-2 cells. ^**^P < 0.01 versus the siCtrl group (n=5). (H) Colocalization of METTL3 protein, IGF2BP3 protein and β-catenin mRNA in renal tubules at 11 days after UIRI. Kidney serial sections from UIRI mice were detected for β-catenin mRNA using *in situ* hybridization (ISH), and immunohistochemical staining for METTL3 and IGF2BP3. Arrows indicate METTL3, IGF2BP3 and β-catenin mRNA colocalization in the same tubule. Scale bar, 50 µm. (F) Overexpression of METTL3 promoted β-catenin mRNA stability in HK-2 cells. The levels of β-catenin mRNA were analyzed at different time points after actinomycin D treatment. ^*^P < 0.05 versus the pcDNA3 group (n=3).

To test this hypothesis, we reanalyzed relevant datasets from Gene Expression Omnibus (GEO) database. Data mining from a published antibody-based methylated RNA immunoprecipitation throughput sequencing (MeRIP-seq, GSE182607) performed in HEK293T cells suggests that the m^6^A levels of 1247 transcripts were downregulated after knocking out METTL3 (*P* < 0.05 and |log_2_FoldChange| > 3). In addition, a published RNA immunoprecipitation throughput sequencing (RIP-seq, GSE90639) in HEK293T cells revealed that 3122 transcripts may be the potential targets of IGF2BP3 (*P* < 0.05 and |log_2_FoldChange| > 3). As shown in Figure 5A, the intersection of these two datasets identified 226 overlapping genes, which included *CTNNB1*. These overlapping genes were then subjected to Gene Ontology (GO) and Kyoto Encyclopedia of Genes and Genomes (KEGG) analyses (Figure 5B). Both the GO biological processes (BP) and KEGG enrichment of overlapping genes pointed to Wnt/β-catenin signaling pathway. Therefore, we chose β-catenin for further studies, as it is the central intracellular mediator of the Wnt signaling 39.

An online m^6^A sites prediction tool SRAMP (http://www.cuilab.cn/sramp) was further used to predict the presence of m^6^A sites in β-catenin mRNA sequence. As presented in Supplementary Table S4, SRAMP detected a total of 15 m^6^A sites in β-catenin mRNA, among which 3 were identified to be with very high confidence (Figure 5C). To validate these m^6^A sites, we performed the MeRIP-qPCR assay (Figure 5D). When METTL3 was knocked down in HK-2 cells, only the m^6^A site 3 from the three predicted regions showed a decrease of the m^6^A methylated β-catenin mRNA (Figure 5E), whereas the β-catenin m^6^A methylation was upregulated when METTL3 was over-expressed in HK-2 cells (Figure 5F). Collectively, these results suggest that β-catenin is a potential target of METTL3-mediated m^6^A modification.

Figure 5G shows that downregulation of METTL3 in HK-2 cells resulted in a decreased steady-state level of β-catenin mRNA, suggesting that m^6^A methylation might play a role in regulating its stability. As previous studies have shown that IGF2BP3 controls β-catenin mRNA stability 20, we next examined the relationship among METTL3, IGF2BP3 and β-catenin mRNA in the fibrotic kidney. As shown in Figure 5H, colocalization of METTL3 and IGF2BP3 proteins with β-catenin mRNA was observed in renal tubular epithelium of the fibrotic kidney after UIRI. Furthermore, overexpression of METTL3 increased the stability of β-catenin mRNA and slowed down its degradation *in vitro* (Figure 5I), suggesting that m^6^A modification stabilizes β-catenin mRNA. Taken together, these results suggest that METTL3 triggers m^6^A modification of β-catenin mRNA, which is then recognized by IGF2BP3, leading to its stabilization and upregulation.

### METTL3 promotes renal fibrotic response by activating β-catenin signaling

**Figure 6:**
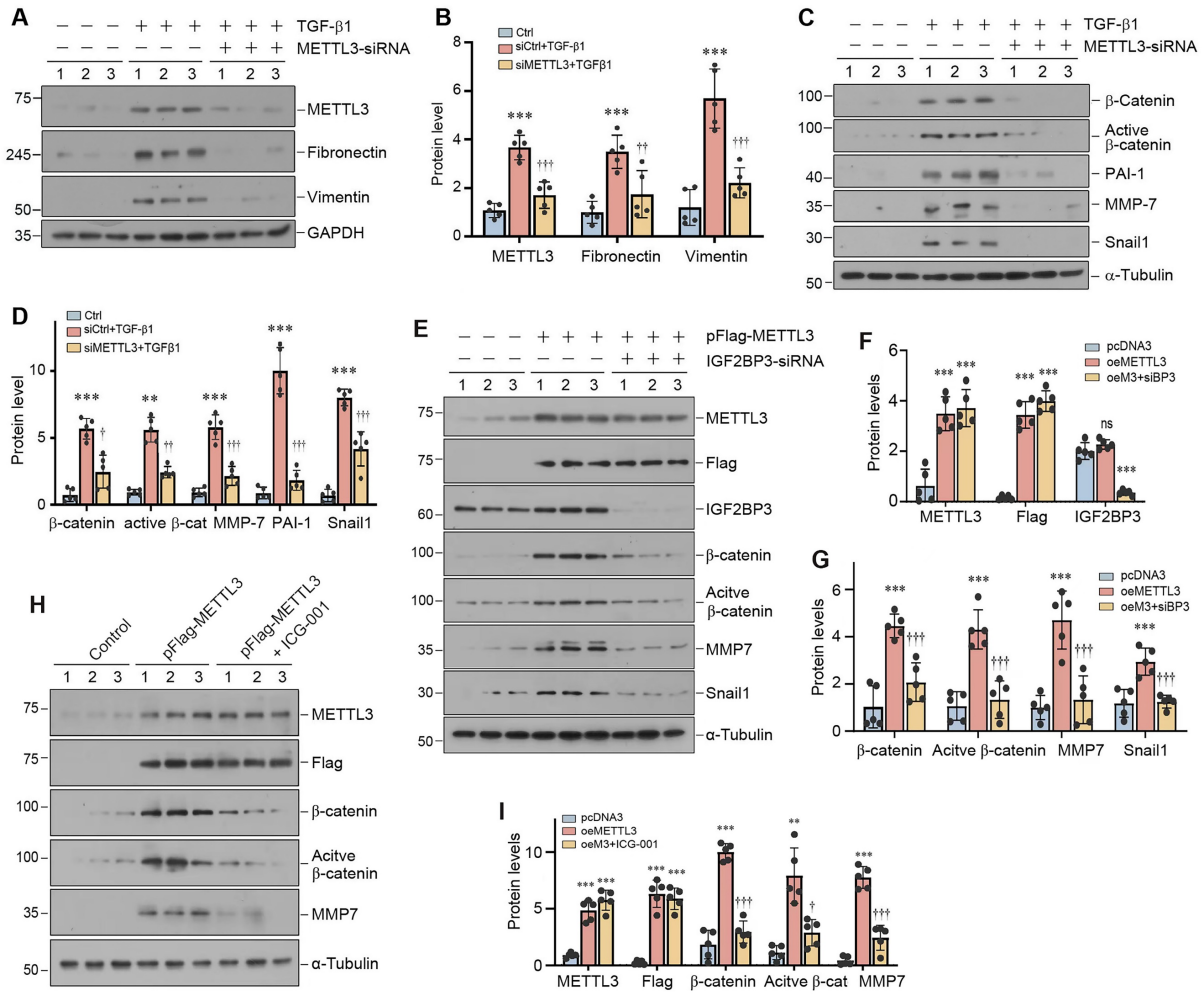
** METTL3 is required for TGF-β1-mediated fibrotic response and β-catenin activation in kidney tubular epithelial cells.** (A, B) Western blots (A) and densitometric quantification (B) demonstrate METTL3, fibronectin and vimentin expression in TGF-β1-stimulated HK-2 cells in the absence or presence of METTL3 siRNA. ^***^P < 0.001 versus controls, ^††^*P* < 0.01, ^†††^*P* < 0.001 versus TGF-β1 plus control siRNA (n = 5). (C, D) Western blots (C) and quantitative data (D) show the expression of β-catenin, active β-catenin, PAI-1, MMP-7 and Snail1 proteins in different groups as indicated. ^**^P < 0.01, ^***^P < 0.001 versus controls; ^††^*P* < 0.01, ^†††^*P* < 0.001 versus TGF-β1 plus control siRNA (n = 5). (E - G) Western blots (E) and quantitative data (F, G) show the expression of METTL3, Flag, IGF2BP3, β-catenin, active β-catenin, PAI-1, MMP-7 and Snail1 proteins in different groups as indicated. ^***^P < 0.001 versus controls; ^†††^*P* < 0.001 versus HK-2 cells overexpress METTL3 (n = 5). (H, I) Western blots (H) and quantitative data (I) show the expression of METTL3, Flag, β-catenin, active β-catenin and MMP-7 proteins in different groups as indicated. ^**^P < 0.01, ^***^P < 0.001 versus controls; ^†^*P* < 0.05, ^†††^*P* < 0.001 versus HK-2 cells overexpress METTL3 (n = 5).

Since TGF-β1 increased m^6^A RNA modification (Figure 1L and M) and induced METTL3 expression (Supplementary Figure S1B and C) in HK-2 cells, we decided to utilize this *in vitro* system to investigate the mechanism of m^6^A modification in kidney fibrogenesis. HK-2 cells were subjected to transfection with control or METTL3-siRNA, followed by incubation with TGF-β1. As presented in Figure 6A and B, TGF-β1-induced METTL3 expression was largely abolished by transfection of METTL3-siRNA. Interestingly, silencing METTL3 inhibited the TGF-β1-induced expression of fibronectin and vimentin in HK-2 cells. We also found that knockdown of METTL3 abolished β-catenin activation and the expression of its downstream target genes such as plasminogen activator inhibitor-1 (PAI-1), matrix metalloproteinase-7 (MMP-7), and transcription factor Snail1 (Figure 6C and D).

To further corroborate the role of IGF2BP3 and β-catenin in mediating METTL3's action, we overexpressed METTL3 or/and knocked down IGF2BP3 expression in HK-2 cells. As shown in Figure 6E and F, the efficiency of METTL3 overexpression and IGF2BP3 knockdown was confirmed. We found that knockdown of IGF2BP3 abolished the induction of β-catenin, active β-catenin, MMP-7 and Snail1 expression stimulated by METTL3 overexpression. Similarly, blockade of β-catenin signaling by a small molecule inhibitor ICG-001 also abolished β-catenin activation and MMP-7 expression induced by METTL3 overexpression (Figure 6H and I). These data indicate a crucial role of IGF2BP3/β-catenin in mediating the profibrotic actions of METTL3 in the kidney.

### Proximal tubule-specific ablation of METTL3 inhibits β-catenin signaling

**Figure 7:**
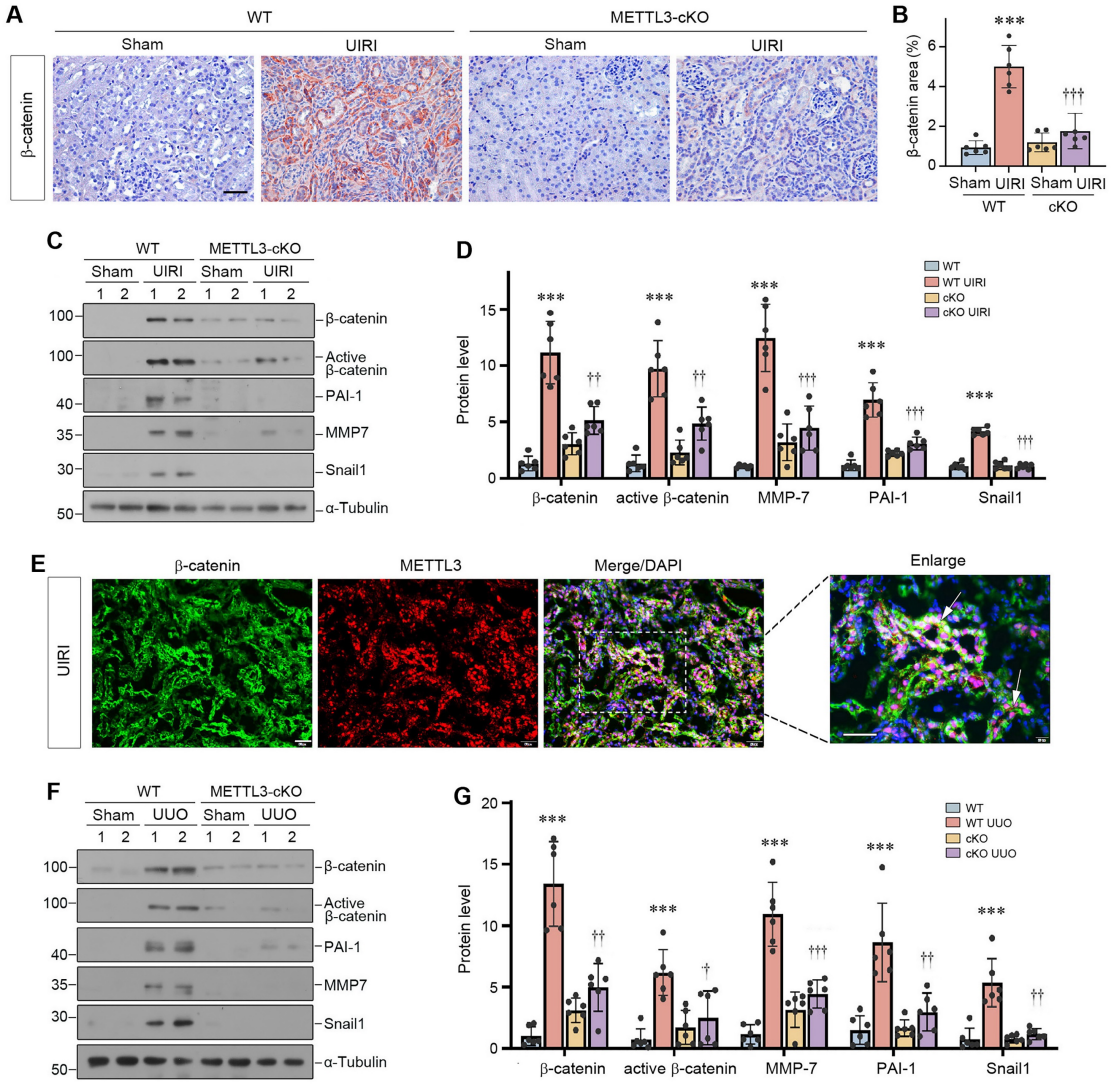
** Proximal tubule-specific ablation of METTL3 abolishes β-catenin activation in the fibrotic kidney after UUO and UIRI.** (A, B) Representative micrographs (A) and quantitative data (B) show the expression of β-catenin at 11 days after UIRI in different groups as indicated (n=6). ^***^P < 0.001 versus Sham group; ^†††^*P* < 0.001 versus WT UIRI group. Scale bar, 50 µm. (C, D) Representative Western blots (C) and quantitative data (D) show protein expression of β-catenin, active β-catenin, PAI-1, MMP-7 and Snail1 in different groups as indicated (n = 6). ^***^P < 0.001 versus Sham group; ^††^*P* < 0.01, ^†††^*P* < 0.001 versus WT UIRI group. (E) Colocalization of METTL3 (red) and β-catenin protein (green) in renal tubules at 11 days after UIRI. Arrows indicate METTL3 and β-catenin colocalization in the same tubules. Scale bar, 50 µm. (F, G) Representative Western blots (F) and quantitative data (G) show protein expression of β-catenin, active β-catenin, PAI-1, MMP-7, and Snail1 in different groups as indicated (n = 6). ^***^P < 0.001 versus Sham group; ^†^*P* < 0.05, ^††^*P* < 0.01, ^†††^*P* < 0.001 versus WT UUO group.

We further investigated the impact of METTL3 on β-catenin signaling in the fibrotic kidney *in vivo*. Immunohistochemical staining showed that renal β-catenin was markedly induced, predominantly in renal tubular epithelium, after UIRI (Figure 7A and B). However, conditional knockout of METTL3 abolished β-catenin expression in the injured kidney (Figure 7A and B). We also examined the expression of β-catenin downstream genes in the fibrotic kidney after UIRI. As shown in Figure 7C and D, UIRI induced β-catenin, active β-catenin, PAI-1, MMP-7 and Snail1 in the kidney of WT mice, whereas proximal tubule-specific ablation of METTL3 abolished the induction of these proteins. Co-immunofluorescence staining also confirmed the colocalization of METTL3 and β-catenin proteins in the fibrotic kidney after UIRI (Figure 7E). Similar results were obtained when WT and METTL3 cKO mice were subjected to UUO (Figure 7F and G). Therefore, it appears clear that METTL3-mediated m^6^A modification is required for β-catenin activation in the fibrotic kidney induced by UIRI or UUO.

**Figure 8:**
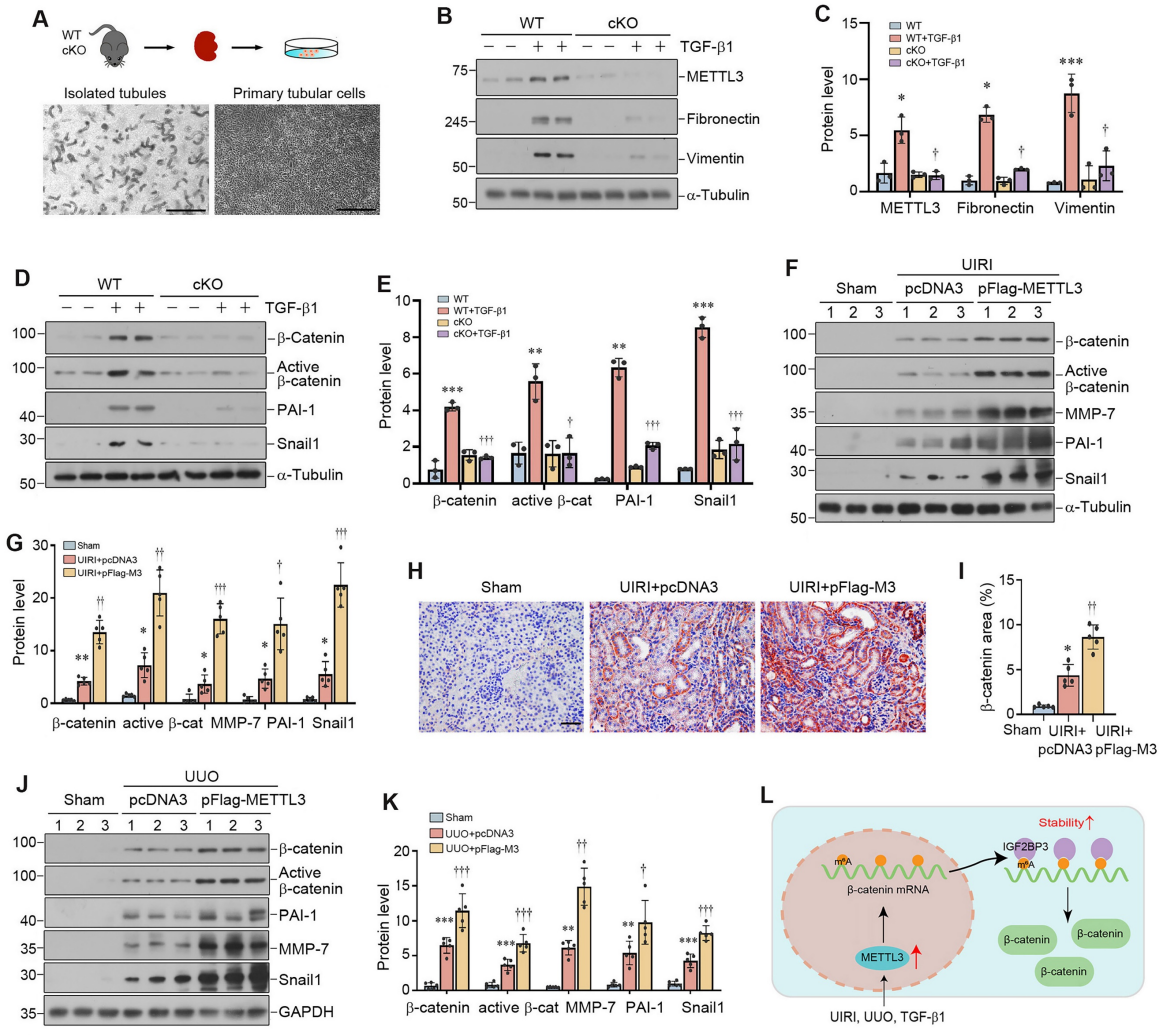
** Overexpression of METTL3 promotes β-catenin signaling *in vivo*.** (A) Establishment of primary kidney tubular epithelial cells from WT and cKO mice. Micrographs show the isolated kidney tubules and cultured primary tubular epithelial cells, respectively. Scale bar, 100 µm. (B, C) Representative Western blots (B) and quantitative data (C) show protein expression of METTL3, fibronectin and vimentin after TGF-β1 treatment in mouse primary tubular epithelial cells (n = 3). ^*^P < 0.05, ^***^P < 0.001 versus WT controls; ^†^P < 0.05 versus WT+TGF-β1. (D, E) Western blots (D) and quantitative data (E) show the expression of β-catenin, active β-catenin, PAI-1 and Snail1 proteins in different groups as indicated (n = 3). ^**^P < 0.01, ^***^P < 0.001 versus WT controls; ^†^*P* < 0.05, ^†††^*P* < 0.001 versus WT+TGF-β1. (F, G) Representative Western blot (F) and quantitative data (G) show the expression of β-catenin, active β-catenin, MMP-7, PAI-1 and Snail1 in different groups as indicated. ^*^P < 0.05, ^**^P < 0.01 versus Sham group; ^†^*P* < 0.05, ^††^*P* < 0.01, ^†††^*P* < 0.001 versus UIRI injected with pcDNA3 group (n=5). (H, I) Representative micrographs (H) and quantitative data (I) show the expression of β-catenin at 11 days after UIRI in different groups (n=5). ^*^P < 0.05 versus Sham group; ^††^*P* < 0.01 versus UIRI injected with pcDNA3 group. Scale bar, 50 µm. (J, K) Representative Western blot (J) and quantitative data (K) show the expression of β-catenin, active β-catenin, MMP-7, PAI-1, and Snail1 in different groups as indicated. ^**^P < 0.01, ^***^P < 0.001 versus Sham group; ^†^*P* < 0.05, ^††^*P* < 0.01, ^†††^*P* < 0.001 versus UUO injected with pcDNA3 group (n=5). (L) Diagraph shows the potential mechanism and pathway in which METTL3 drives kidney fibrosis. Kidney injury such as UIRI, UUO or TGF-β induced METTL3 expression, which promotes β-catenin mRNA m^6^A methylation. The m^6^A-methylated β-catenin mRNA is then recognized and bound by the reader protein IGF2BP3, which leads to its stabilization, induction and activation, thereby promoting kidney fibrosis.

We further isolated primary tubular epithelial cells (PTECs) from the WT and cKO mice (Figure 8A). As shown in Figure 8B and C, knockout of METTL3 blocked TGF-β1-induced vimentin and fibronectin expression in primary tubular cells. Consistently, knockout of METTL3 also abolished β-catenin activation and its downstream PAI-1 and Snail1 expression induced by TGF-β1 (Figure 8D and E). Collectively, these findings demonstrate that METTL3 promotes renal fibrotic response by activating β-catenin signaling.

### Overexpression of METTL3 promotes β-catenin signaling *in vivo*

To provide additional evidence for METTL3 and β-catenin connection, we examined the potential role of METTL3 in activating β-catenin in the fibrotic kidney *in vivo*. As shown in Figure 8F and G, renal expression of β‐catenin and its downstream proteins, such as active β-catenin, PAI-1, MMP-7 and Snail1, were induced in the fibrotic kidney after UIRI, and overexpression of METTL3 further aggravated the induction of these proteins. Immunostaining for β-catenin gave rise to similar results (Figure 8H and I), suggesting that METTL3 contributes to the activation of β-catenin signaling after UIRI.

We also confirmed these findings in mouse model of UUO. As shown in Figure 8J and K, overexpression of METTL3 could further upregulate renal β-catenin, active β-catenin, MMP-7, PAI-1 and Snail1 expression after UUO. Together, these results suggest that METTL3-mediated m^6^A modification plays a crucial role in promoting kidney fibrosis by targeting β-catenin mRNA and preventing its degradation via an IGF2BP3-dependent mechanism (Figure 8L).

## Discussion

The m^6^A methylation is the most common modification in eukaryotic mRNA and plays a critical role in the pathogenesis of diverse human diseases, including tumorigenesis, heart failure, diabetes, acute kidney injury and polycystic kidney disease 40-42. However, its role in kidney fibrosis was largely elusive and somewhat controversial. In this study, we showed that kidney fibrosis is associated with an upregulation of overall m^6^A methylated RNA level in the fibrotic kidney. By generating proximal tubule-specific conditional METTL3 knockout mice, we demonstrate that blockade of METTL3-mediated m^6^A modification inhibits kidney fibrosis, whereas overexpression of METTL3 promotes fibrosis progression in CKD models. Mechanistically, METTL3 promotes kidney fibrosis by enhancing the m^6^A methylation of β-catenin mRNA, which is then recognized by IGF2BP3, leading to an increased β-catenin mRNA stability. These studies intuitively link the m^6^A modification to the induction and activation of β-catenin, a master intracellular mediator that controls a wide variety of pro-fibrotic genes in kidney fibrogenesis 43. Our findings also underscore that the posttranscriptional regulation of β-catenin at the mRNA level, which is often under-appreciated, plays a pivotal role in controlling Wnt/β-catenin signaling in the fibrotic kidney.

The present study provides unambiguous evidence that kidney fibrosis is a state of hyperactive m^6^A RNA methylation. Currently, studies on the status of m^6^A methylation in CKD are limited and somewhat controversial 44-46. While two studies show that the total m^6^A methylated RNA level is increased in mouse kidney after UUO 45, 46, another report indicates a time-dependent decrease in total m^6^A methylated RNA levels 44. The reason behind this discrepancy is unknown, but it highlights that more comprehensive investigation is required to validate the status of m^6^A methylation in this setting. In the present study, two models of kidney fibrosis induced by UUO and UIRI or cultured tubular HK-2 cells stimulated with TGF-β1 *in vitro* confirm that the overall m^6^A methylated RNA level is markedly upregulated during the evolution of renal fibrosis (Figure 1). This is supported by an induction of METTL3 in both animal models and human kidney biopsies from CKD patients (Figure 1 and 2). More importantly, proximal tubule-specific ablation of METTL3 protects mice against developing kidney fibrosis (Figure 3), indicating that hyperactive m^6^A methylation is detrimental promoting fibrotic lesions. Taken together, it is reasonable to conclude that kidney fibrosis is associated with the upregulation of overall m^6^A RNA methylation.

Kidney fibrosis is associated with induction of many methyltransferases such as METTL3, METTL14, WTAP and METTL16 (Figure 1). Reanalysis of an independent RNA-seq dataset also reveals that almost all methyltransferases are upregulated in the obstructed kidney after UUO in a time-dependent fashion (Figure 1K), consistent with the notion of an increased overall m^6^A methylation. We chose METTL3 for detailed studies, because it is the most upregulated in the fibrotic kidneys (Figure 1). In addition, METTL3 is the key catalytic subunit of the methyltransferase complex and its role in regulating fibrotic diseases is established in liver, lung and heart 47-49. Furthermore, METTL3 upregulation is observed in kidney biopsies from CKD patients and correlates with the degree of kidney pathology (Figure 2), suggesting its clinical relevance. Intriguingly, conditional knockout of METTL3 alleviates kidney fibrosis induced by UUO and UIRI in mice, underscoring the importance and significance of METTL3 in kidney fibrosis. However, it should be pointed out that besides METTL3, other methyltransferases, which are upregulated in CKD as well, may also play a role in kidney fibrosis. In this regard, recent studies show that METTL14 is increased in DKD, and knockdown of METTL14 protects against streptozotocin-induced kidney fibrosis in mice 50. Furthermore, WTAP and METTL16 have been shown to participate in regulating cardiac fibrosis and chronic hepatitis B fibrosis, respectively 51, 52. Future studies on the role of these methyltransferases in kidney fibrogenesis are warranted.

One interesting finding of the present study is the identification of β-catenin mRNA as a target of METTL3-mediated m^6^A modification. This conclusion is reached through a combination of bioinformatics analysis and experimental validation. Of note, m^6^A-modified mRNAs can be recognized by IGF2BP family proteins, including IGF2BP1, IGF2BP2 and IGF2BP3 21. We recently have shown that IGF2BP3 has the capability to directly interact with β-catenin mRNA and increases its stability 20. These observations led us to speculate a potential connection among METTL3, IGF2BP3, β-catenin mRNA in the context of m^6^A modification. Indeed, data mining led to the identification of β-catenin mRNA as a target of the m^6^A modification (Figure 5). This finding is validated by the MeRIP-qPCR assay. Furthermore, co-localization of METTL3, IGF2BP3, and β-catenin mRNA is evident in renal tubular epithelium of the fibrotic kidney (Figure 5). As both METTL3 (Figure 1) and IGF2BP3 are markedly upregulated in the fibrotic kidney 20, this would cause m^6^A methylated β-catenin mRNA to be recognized by IGF2BP3, leading to its stabilization. Consistently, either knockdown of IGF2BP3 or inhibition of β-catenin signaling abolishes the fibrogenic actions of METTL3 (Figure 6).

The connection of METTL3/IGF2BP3/β-catenin mRNA suggests a novel route leading to β-catenin activation without the involvement of Wnt ligands. Extensive studies show that β-catenin plays a crucial role in driving kidney fibrogenesis through its regulation of numerous fibrosis-related genes, such as PAI-1, MMP-7 and Snail1 39. It is widely presumed that β-catenin is principally regulated at the posttranslational level through phosphorylation and ubiquitin-mediated degradation 19, 20, 43. Herein, we provide evidence that mRNA m^6^A modification is another layer of regulation of β-catenin expression. As the proximal tubule-specific ablation of METTL3 almost completely abolished renal β-catenin expression after injury in cKO mice (Figure 7), this suggests that the METTL3-mediated m^6^A modification plays a predominant role in triggering β-catenin activation in fibrotic kidney. The discovery of this previously unrecognized route of β-catenin regulation via m^6^A methylation by METTL3/IGF2BP3 may present a novel opportunity for developing therapeutic interventions for CKD patients.

There are several questions remaining to be addressed. One of the unresolved issues is whether METTL3 regulates kidney fibrosis by promoting m^6^A methylation of other target transcripts. In view of that many RNA transcripts are subjected to m6A modification, we cannot exclude the possibility that other targets and pathways are also involved in mediating METTL3-induced kidney fibrosis. Along this line, recent studies suggest that METTL3 enhances the m^6^A modification of Ena/VASP-like (EVL) mRNA to improve its stability and expression in an IGF2BP2-dependent manner 53. Another issue is that as the m^6^A modification is reversible and can be demethylated by two known erasers, α-ketoglutarate-dependent dioxygenase AlkB homolog 5 (ALKBH5) and fat mass and obesity-associated protein (FTO) 54, whether these erasers participate in the regulation of m^6^A modification in fibrotic kidney remains to be determined. Of interest, both ALKBH5 and FTO are downregulated in the early stage of kidney fibrosis after UUO (Supplementary Figure S1). Undoubtedly, more studies are needed in this area in the future.

In summary, the present study reveals that METTL3 is induced in the fibrotic kidney and promotes the m^6^A methylation of β-catenin mRNA, which is then recognized by IGF2BP3, leading to its stabilization. These findings uncover a novel mechanism leading to β-catenin activation in the evolution of kidney fibrosis and pave a new avenue for developing effective therapeutic strategies for CKD.

## Supplementary Material

Supplementary figures and tables.

## Abbreviations

CKD: chronic kidney disease
CTIN: chronic tubulointerstitial nephritis
DKD: diabetic kidney disease
FSGS: focal and segmental glomerulosclerosis
IgAN: IgA nephropathy
IGF2BP3: insulin-like growth factor 2 mRNA binding protein 3
ISH: *in situ* hybridization
LN: lupus nephritis
m6A: N6-methyladenosine
MeRIP: methylated RNA immunoprecipitation
METTL3: methyltransferase-like 3
METTL14: methyltransferase-like 14
MN: membranous nephropathy
α-SMA: α-smooth muscle actin
RT-qPCR: reverse transcription quantitative real-time PCR
UIRI: unilateral ischemia-reperfusion injury
UUO: unilateral ureteral obstruction
WTAP: Wilms tumor 1 associated protein
