# Supplementary material for: m6A RNA methylation drives kidney fibrosis by upregulating β-catenin signaling
Source: Int J Biol Sci. 2024 Jun 3;20(8):3185–200. doi: 10.7150/ijbs.96233 (PMC11186362; doi:10.7150/ijbs.96233)
Supplement: Supplementary file 1 — Supplementary figures and tables. [file ijbsv20p3185s1.pdf]

m6A RNA methylation drives kidney fibrosis by upregulating β-catenin signaling

Yinyi Long, Dongyan Song, Liuyan Xiao, Yadie Xiang, Dier Li, Xiaoli Sun, Xue Hong, Fan Fan Hou, Haiyan Fu, Youhua Liu

Supplemental Figures

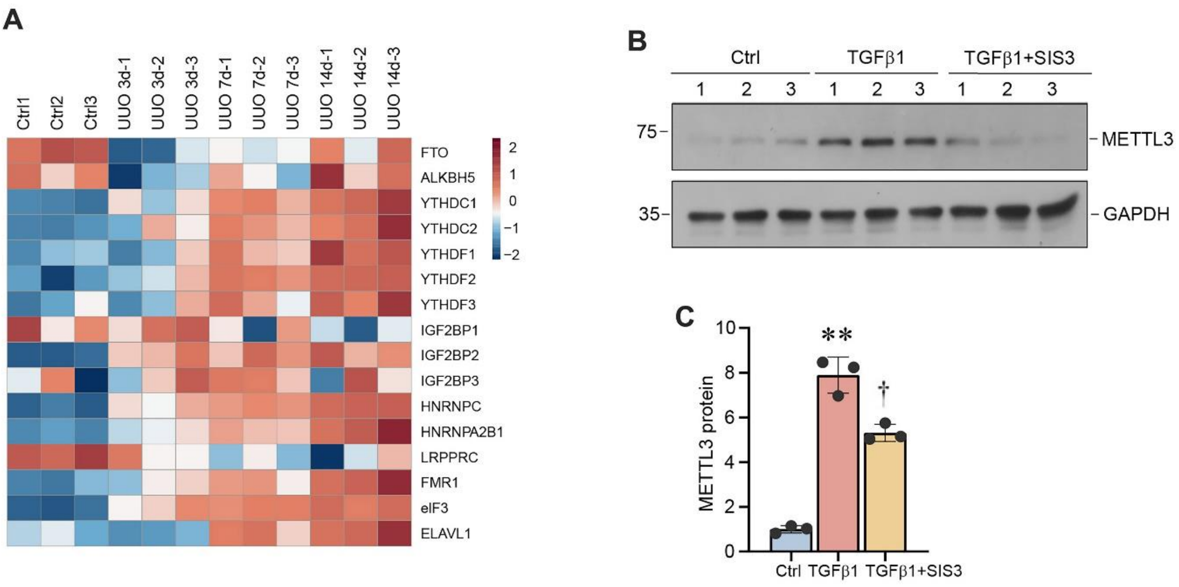

Figure S1. The expression of m6A readers and erasers in the kidney at different time points after UUO. (**A**) Re-analysis of the RNA-seq data (GSE118339) demonstrated the expression and dynamic of m6A readers and erasers in control and UUO kidneys at different time points (day 3, 7, 14) after surgery. Red indicates a higher abundance, while blue indicates a lower abundance. (**B**, **C**) Induction of METTL3 by TGF-β1 in kidney tubular epithelial cells (HK2) is dependent on Smad3 signaling. Representative Western blot (**B**) and quantitative data (**C**) showed that Smad3 inhibitor SIS3 abolished the induction of METTL3 proteins in TGF-β1-stimulated HK2 cells. $^{**}P < 0.01$ versus controls (n=3), $^{\dagger}P < 0.05$ versus TGFβ1 group (n=3).

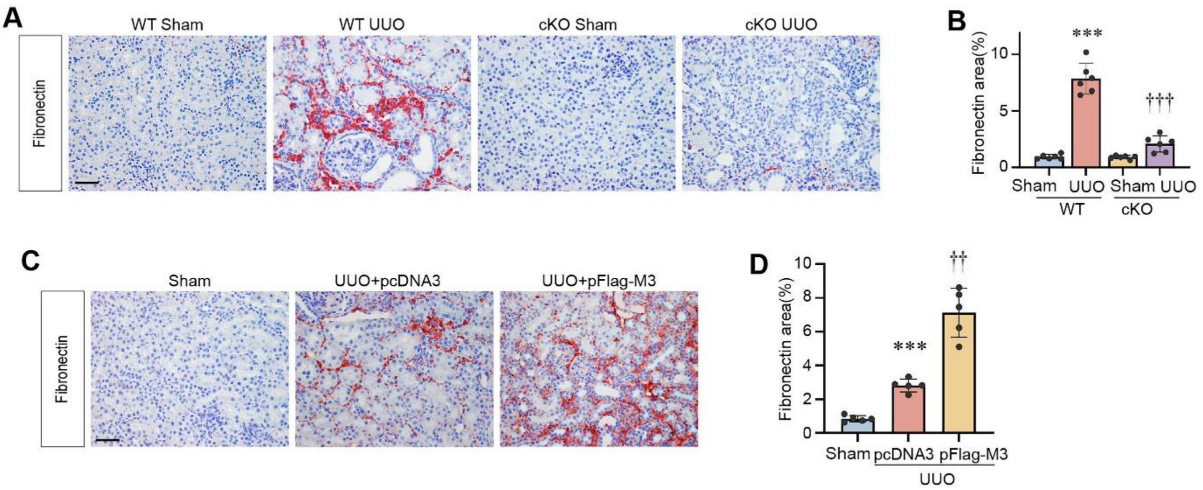

Figure S2. Proximal tubule-specific knockout or overexpression of METTL3 affects renal fibronectin expression. (A, B) Proximal tubule-specific conditional knockout of METTL3 inhibits renal fibronectin expression after UUO. Representative micrographs (A) and semi-quantification (B) show fibronectin expression by immunohistochemical staining at 7 days after UUO in different groups. $^{***}P < 0.001$ versus Sham group; $^{\dagger\dagger\dagger}P < 0.001$ versus WT UUO group (n=6). Scale bar, 50 μm. (C, D) Overexpression of METTL3 aggravates fibronectin expression after UUO. Representative micrographs (C) and semi-quantification (D) show renal expression of fibronectin at 7 days after UUO in different groups. $^{***}P < 0.001$ versus Sham group; $^{\dagger\dagger}P < 0.01$ versus UUO injected with pcDNA3.1 group (n=5). Scale bar, 50 μm.

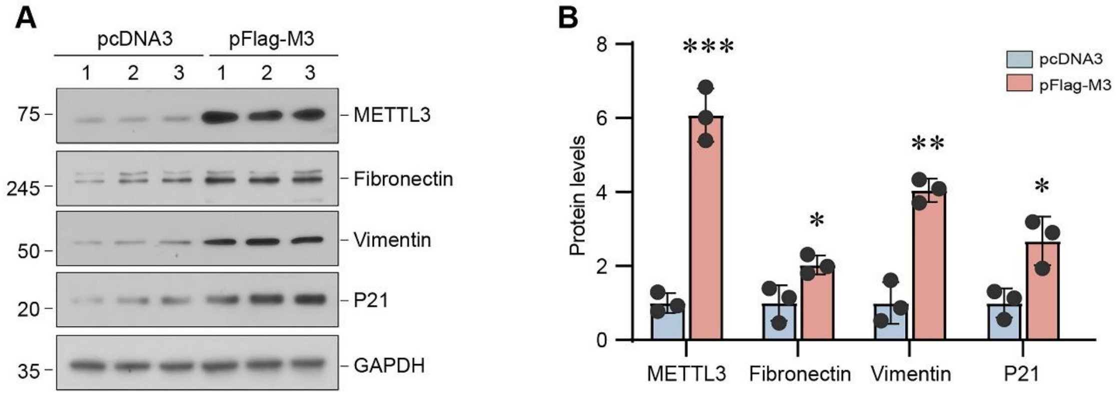

Figure S3. Overexpression of METTL3 in HK2 cells promote fibrotic response. (A-B) Representative Western blot (A) and quantitative data (B) showed that overexpression of METTL3 induced the upregulation of fibronectin, vimentin and p21 expression in HK2 cells. $^{*}P < 0.05$, $^{**}P < 0.01$, $^{***}P < 0.001$ versus pcDNA3 (n=3).

Table S1. The sources of antibodies used in this study.

Antibodies	Catalogue number	Company	Usage	Location
**Primary antibodies**				
anti-METTL3	Ab195352	Abcam	WB/IHC/IF	Cambridge, MA
anti-METTL14	26158-1-AP	Proteintech	WB	Rosemont, IL
anti-WTAP	60188-1-lg	Proteintech	WB	Rosemont, IL
anti-IGF2BP3	Ab179807	Abcam	IHC	Cambridge, MA
anti-fibronectin	F3648	Sigma-Aldrich	WB/IHC	St. Louis, MO
anti-α-SMA	A2547	Sigma-Aldrich	WB/IHC	St. Louis, MO
anti-collagen I	BA0325	Boster Biological Technology	WB	Wuhan, China
anti-vimentin	Ab92547	Abcam	WB	Cambridge, MA
anti-β-catenin	610154	BD Biosciences	WB/IF	San Jose, CA
anti-β-catenin	Ab15180	Abcam	IHC	Cambridge, MA
anti-active β-catenin	#19807	Cell Signaling Technology	WB	Danvers, MA
anti-MMP-7	GTX104658	GeneTex	WB	Irvine, CA
anti-PAI-1	AF3828	R & D Systems	WB	Minneapolis, MN
anti-snail 1	Ab180714	Abcam	WB	Cambridge, MA
anti-flag	F1804	Sigma-Aldrich	WB/IHC	St. Louis, MO
anti-α-tubulin	RM2007	Ray Antibody Biotech	WB	Peachtree Corners, GA
anti-GAPDH	RM2002	Ray Antibody Biotech	WB	Peachtree Corners, GA
anti-p21	A2691	ABclonal	WB	Wuhan, China
**Secondary antibodies**				
Goat anti-mouse	BA1050	Boster Biological Technology	WB	Wuhan, China
Goat anti-rabbit	BA1054	Boster Biological Technology	WB	Wuhan, China
Rabbit anti-goat	BA1060	Boster Biological Technology	WB	Wuhan, China
Donkey Anti-Mouse	715-0165-150	Jackson ImmunoResearch	IHC	West Grove, PA
Donkey Anti-Rabbit	711-065-152	Jackson ImmunoResearch	IHC	West Grove, PA
Donkey Anti-Mouse	715-225-150	Jackson ImmunoResearch	IF	West Grove, PA
Donkey Anti-Rabbit	711-1165-152	Jackson ImmunoResearch	IF	West Grove, PA

Table S2. The sequences of primers used for qRT-PCR in this study.

Gene	Species	Primer Sequence 5’ to 3’	
		**Forward**	**Reverse**
CTNNB1	Human	GGTACTGACTTTGCTTGCTT	CATGAAATAGATCCACCTGC
GAPDH	Human	GGAGCGAGATCCCTCCAAAAT	GGCTGTTGTCATACTTCTCATGG
mettl3	Mouse	TGACCACTCCAAGCTGTCTG	TCTCCAGATCAACATCGGAG
Mettl14	Mouse	TGGTCGGGAAAGAAACCGAT	GGGAGTGAGCTCTGAAGCAA
Mettl16	Mouse	GCCTGCGAGATGGCTCTAA	AAGACTTACTCTCCCATTCAGATT
wtap	Mouse	CTCAAGCAAGTTCAGCAGCC	TCAGGCGTAAACTTCCAGGC
Ctnnb1	Mouse	ACTTGCCACACGTGCAATTC	ATGGTGCGTACAATGGCAGA
Gapdh	Mouse	TGACCTCAACTACATGGTCTACA	CTTCCCATTCTCGGCCTTG

Table S3. Demographic and clinical data of CKD patients.

**Characteristics**	**CKD patients**
**Gender-No. (%)**	
Male	14 (56)
Female	11 (44)
**Age at entry-years**	
Mean ± SEM	43.2±17.1
Range	18-77
**Time from diagnosis to biopsy (month)**	12.0 (3.5-24.0)
**Scr (μmol/l)**	133.7±61.3
**eGFR (ml/min/1.73m^2)**	57.5±27.9
**Pathological Diagnosis-No. (%)**	
DN	4 (16)
CTIN	4 (16)
LN	4 (16)
IgAN	5 (20)
FSGS	3 (12)
MN	5 (20)

Table S4. The predicted 15 m^{6}A residues located across β-catenin sequence according to online SRAMP database

	Position	Sequence context	Score (binary)	Score (knn)	Score (spectrum)	Score (combined)	Decision
1	134	ACCGCAGGTCGAGGACGGTCGGACTCCCGCGGCGGGAGGAGCCTG	0.69	0.755	0.545	0.63	m6A site (High confidence)
2	309	TGGCAGCAACAGTCTTACCTGGACTCTGGAATCCATTCTGGTGCC	0.706	0.729	0.586	0.659	m6A site (High confidence)
3	466	AACAAGTAGCTGATATTGATGGACAGTATGCAATGACTCGAGCTC	0.641	0.682	0.517	0.593	m6A site (Moderate confidence)
4	518	ACGAGCTGCTATGTTCCCTGAGACATTAGATGAGGGCATGCAGAT	0.798	0.334	0.818	0.782	m6A site (Very high confidence)
5	679	TTGCCACACGTGCAATCCCTGAACTGACAAAACTGCTAAATGACG	0.684	0.727	0.434	0.586	m6A site (Moderate confidence)
6	1315	CTGGTGGAATGCAAGCTTTAGGACTTCACCTGACAGATCCAAGTC	0.687	0.690	0.553	0.633	m6A site (High confidence)
7	1353	CCAAGTCAACGTCTTGTTCAGAACTGTCTTTGGACTCTCAGGAAT	0.688	0.546	0.542	0.622	m6A site (High confidence)
8	1364	TCTTGTTCAGAACTGTCTTTGGACTCTCAGGAATCTTTCAGATGC	0.654	0.746	0.525	0.607	m6A site (Moderate confidence)
9	1424	AGGGATGGAAGGTCTCCTTGGGACTCTTGTTCAGCTTCTGGGTTC	0.673	0.705	0.432	0.578	m6A site (Low confidence)
10	1684	ATGCAGTTCGCCTTCACTATGGACTACCAGTTGTGGTTAAGCTCT	0.697	0.705	0.566	0.645	m6A site (High confidence)
11	1990	ACAACCGAATTGTTATCAGAGGACTAAATACCATTCCATTGTTTG	0.618	0.640	0.458	0.555	m6A site (Low confidence)
12	2305	GGAATGAGACTGCTGATCTTGGACTTGATATTGGTGCCCAGGGAG	0.668	0.530	0.541	0.610	m6A site (Moderate confidence)
13	2422	TGGGTATGGACCCCATGATGGAACATGAGATGGGTGGCCACCACC	0.792	0.405	0.830	0.788	m6A site (Very high confidence)
14	2891	TTTATACAGCTGTATTGTCTGAACTTGCATTGTGATTGGCCTGTA	0.604	0.449	0.512	0.559	m6A site (Low confidence)
15	3181	AAGAATATCTGTAATGGTACTGACTTTGCTTGCTTTGAAGTAGCT	0.86	0.151	0.764	0.786	m6A site (Very high confidence)
